# Diagnostic flowchart to estimate the morphology of left descending artery lesions by initial electrocardiogram in ST‐elevation myocardial infarction

**DOI:** 10.1111/anec.12695

**Published:** 2019-09-25

**Authors:** Toshiharu Fujii, Misaki Hasegawa, Norihito Nakamura, Yuji Ikari

**Affiliations:** ^1^ Department of Cardiology Tokai University School of Medicine Isehara Japan

**Keywords:** diagonal branch, electrocardiography, ST‐elevation myocardial infarction, wrapped left anterior descending artery

## Abstract

**Background:**

ST‐segment deviations in an initial 12‐lead electrocardiogram provide anatomical information in ST‐elevation myocardial infarction (STEMI). A diagnostic flowchart was formulated to estimate the anatomical characteristics of a culprit left anterior descending artery (LAD).

**Methods:**

The present study analyzed 252 STEMI patients whose culprit lesions were confirmed to be LAD as an observational study. LAD morphology, wrapped (*n* = 26) or not (*n* = 226), and the positional relationship to first diagonal branch (*n* = 162 in proximal, *n* = 90 in distal lesions) were assessed. Their ST‐segment deviations and such anatomical characteristics were examined.

**Results:**

Reciprocal ST depression in nonwrapped LAD was frequent in patients without diagonal branch flow (49.3%–18.8% in II, *p* < .01; 66.4%–36.3% in III, *p* < .01; 63.7%–30.0% in aVF, *p* < .01). ST elevation in inferior leads was the characteristics of wrapped LAD but was not the case in patients without diagonal flow (50%–0% in II, 60%–0% in III, and 60%–0% in aVF). ST elevation in lateral leads to the diagonal branch in nonwrapped LAD is more frequent for proximal than distal lesions (36.3% vs. 15.0% in I, *p* < .01; 50.7% vs. 16.3% in aVL, *p* < .01), but this was not observed for wrapped LAD (18.8% vs. 20.0% in I, *p* = .72; 31.3% vs. 10.0% in aVL, *p* = .21). Positive and negative predictive values for the diagnostic accuracy of suggested diagnostic flow based on the above results were 0.794 and 0.478, respectively.

**Conclusions:**

Our suggested diagnostic flowchart provides enough diagnostic accuracy to estimate culprit morphology.

## 1 INTRODUCTION

Electrocardiography has several advantages including not only its simplicity, but also its ability to estimate the location of a myocardial ischemic area. It has played a central role in the diagnosis of ST‐elevation myocardial infarction (STEMI) in an emergency setting, even in an era of modern advanced diagnostic modalities.

Since an ST‐segment deviation can reflect transmural myocardial ischemia, its distribution in a standard 12‐lead electrocardiogram (ECG) provides an important clue in estimating the boundary of the ischemic region and the morphological characteristics of ischemia‐related arteries.

However, patients with a “wrapped left anterior descending artery (LAD),” where a long LAD artery perfuses the inferior wall through the cardiac apex, show a confusing electrocardiographic finding in the distribution of ST‐segment deviations since ischemia in the inferior area is also involved. Moreover, whether the occlusion level involves the major side branches further complicates electrocardiographic interpretation.

The purpose of the present study was to assess the relationship between the distribution of ST‐segment deviation and LAD morphology or major side branches and to design a diagnostic flowchart to estimate the morphology and location of culprit LAD lesions by initial ECG findings in STEMI.

## 2 METHODS

### 2.1 Study design and population

To design a diagnostic flowchart to estimate the morphology and location of culprit LAD lesions by initial ECG in STEMI, the relationship between the distribution of ST‐segment deviations and LAD morphology or major branches was analyzed. The present study was designed as an observational study that surveyed 1,019 consecutive STEMI patients, from January 2006 to April 2018, at Tokai University School of Medicine who met the diagnostic criterion of the fourth universal definition of myocardial infarction (Thygesen et al., 2019) and whose culprit lesions were confirmed to be LAD by emergency coronary angiography within 24 hr of symptom onset. They included 453 patients with culprit LAD lesions. Of these, 201 patients were excluded according to the following exclusion criteria: a history of coronary bypass graft or myocardial infarction, an unanalyzable ECG record, an ECG except for a sinus rhythm, electronic pacing rhythm, ECG showed left or right bundle branch blocks, nonspecific intraventricular conduction delay, thrombolysis in myocardial infarction grade of 2 or 3 for flow in an initial angiogram (Rao et al., 1988), and wrapped or nonwrapped LAD were unable to be distinguished. Finally, 252 patients with LAD lesions were extracted as a target study population.

LAD morphology was assessed and classified as wrapped LAD (*n* = 26) or not (*n* = 226) by emergency coronary angiography. The location of the culprit lesion was assessed in a positional relationship to the first septal and diagonal branches, and proximal or distal lesions to these branches were determined by the study population (*n* = 130 for proximal lesion, and *n* = 122 for distal lesion to the first septal branch: *n* = 162 for proximal, and *n* = 90 for the distal lesion to the first diagonal branch).

The present study was approved by the Institutional Review Board for Clinical Research, Tokai University.

### 2.2 Definitions

A standard 12‐lead resting surface ECG was carried out (paper speed: 25 mm/s, calibration: 1 mV = 10 mm). This initial ECG, which was recorded after hospital arrival and before emergency coronary angiography, was required to meet the following ECG inclusion criteria: sinus rhythm and an analyzable record. A left anterior hemiblock was defined as a qR pattern in lead aVL and a left axis deviation less than −45°. QT intervals were measured from the beginning of the QRS complex to the end of the T wave using the lead with the longest duration, and these were corrected for heart rate according to Bazett's and Fridericia's formula (QTc) (Bazett, 1997).

ST‐elevation myocardial infarction was diagnosed according to the fourth universal definition of myocardial infarction (Thygesen et al., 2019).

ST‐segment deviations, elevations and depressions, were assessed at the J‐point and were determined according to American Heart Association (AHA)/American College of Cardiology Foundation (ACCF)/Heart Rhythm Society (HRS) recommendations: a ST‐segment elevation was defined as ≥0.2 mV in leads V2–3 in men ≥40 years of age, ≥0.25 mV in leads V2–3 in men <40 years of age, ≥0.15 mV in leads V2–3 in women, and ≥0.1 mV in all other leads. A ST‐segment depression was defined as ≥0.05 mV in leads V_2_–V_3_ and ≥0.1 mV in all other leads (Wagner et al., 2009).

A “wrapped LAD” was defined as a LAD from a postreperfusion coronary angiogram that perfused at least one‐fourth of the inferior wall of the left ventricle in a right anterior oblique projection (Sapin, Musselman, Dehmer, & Cascio, 1992; Sasaki, Yotsukura, Sakata, Yoshino, & Ishikawa, 2001; Sonmez et al., 2012).

### 2.3 Statistical analysis

Numerical factors with a skewed distribution are shown as medians (interquartile range), and a Mann–Whitney test was used to determine their statistical difference. A chi‐square test was used to determine differences in categorical variables. Sensitivity, specificity, positive predictive values (PPV), and negative predictive values (NPV) were used to assess diagnostic accuracy. A *p* value < .05 was considered statistically significant. *p* Values in the tables show statistical comparisons. All statistical calculations were performed using JMP version 11 (SAS Institute, Inc.).

## 3 RESULTS

The present study assessed the relationship between the distribution of ST‐segment deviation, and the morphology and location of culprit LAD lesions by initial ECG in STEMI. This led to the design of a diagnostic flowchart to estimate the morphology and location of culprit LAD lesions.

Baseline patients’ characteristics and ECG parameters are summarized in Table 1.

**Table 1:** Patients’ baseline characteristics

	*n* = 252
Age, years	65.0 (55.0, 74.0)
Male, *n*	206 (81.7%)
Currently smoking, *n*	95 (37.7%)
Hypertension, *n*	175 (69.4%)
Dyslipidemia, *n*	190 (75.4%)
Diabetes mellitus, *n*	91 (36.1%)
Hemodialysis, *n*	2 (0.8%)
Hemoglobin, mg/dl	15.0 (13.4, 16.2)
LDL‐chol, mg/dl	129.0 (104.0, 152.0)
HDL‐chol, mg/dl	47.0 (39.0, 55.0)
Triglyceride, mg/dl	104.5 (64.0, 170.0)
Serum creatinine, mg/dl	0.8 (0.7, 1.0)
eGFR, ml min^−1^ 1.73 m^−2^	68.3 (54.6, 81.7)
ECG parameters	
Heart rate, bpm	78.0 (65.0, 92.0)
QRS axis, degree	35.5 (−4.8, 62.0)
QRS duration, ms	98.0 (88.0, 106.0)
LAHB, *n*	17 (6.7%)
QT interval, ms	395.0 (370.0, 420.0)
QTc, Bazett	447.9 (425.4, 468.3)
QTc, Fridericia	429.2 (414.0, 445.9)

Abbreviations: eGFR, estimated glomerular filtration rate; ECG, electrocardiogram; HDL, high high‐density lipoprotein; LAHB, left anterior hemiblock; QTc, corrected QT; LDL, low‐density lipoprotein.

### 3.1 Relationship between ST‐segment deviation and first septal branch flow

The study population was assessed in terms of whether the culprit lesion was located at a site proximal or distal to the first septal branch. The proportion of ST‐segment elevations across 12 leads was compared between these two groups (Table 2). An ST‐segment elevation in leads Ⅰ, aVL, and V_1_ was present in a significantly higher proportion of patients with a proximal lesion than in those with a distal lesion (36.9% vs. 18.0% in Ⅰ, *p* < .01; 51.5% vs. 21.3% in aVL, *p* < .01; 74.6% vs. 49.2% in V_1_, *p* < .01, respectively). Conversely, a proximal lesion was found in a significantly lower proportion of patients with an ST‐segment elevation in leads II and aVF compared to a distal lesion (0% vs. 6.6% in II, *p* < .01; 0.8% vs. 5.7% in aVF, *p* = .03, respectively). When an ST‐segment elevation was observed in all of leads Ⅰ, aVL, and V_1_, the diagnostic accuracy of the lesion location at a site proximal to the first septal branch showed a sensitivity of 0.200, specificity of 0.951, PPV of 0.813, and NPV of 0.527, respectively.

**Table 2:** Relationship between 1st septal branch flow and ST‐segment elevation

ST‐segment elevation, *n*	I	II	III	aVR	aVL	aVF	V_1_	V_2_	V_3_	V_4_	V_5_	V_6_
Proximal lesion to 1st septal br. *n* = 130	48 (36.9%)	0	2 (1.5%)	11 (8.5%)	67 (51.5%)	1 (0.8%)	97 (74.6%)	130 (100%)	130 (100%)	114 (87.7%)	84 (64.6%)	30 (23.1%)
Distal lesion to 1st septal br. *n* = 122	22 (18.0%)	8 (6.6%)	5 (4.1%)	4 (3.3%)	26 (21.3%)	7 (5.7%)	60 (49.2%)	122 (100%)	122 (100%)	112 (91.8%)	84 (68.9%)	39 (32.0%)
*p* Value	<0.01	<0.01	0.22	0.08	<0.01	0.03	<0.01	N/A	N/A	0.28	0.48	0.11

### 3.2 Wrapped LAD and first diagonal branch flow

The study population was divided into those with a wrapped or nonwrapped LAD; moreover, they were assessed on whether the culprit lesion was located at a site proximal or distal to the first diagonal branch. Table 3 shows the number of patients with ST‐segment deviations according to LAD morphology, and the presence or absence of diagonal branch flow. With a nonwrapped LAD, a reciprocal ST‐segment depression in leads II, III, and aVF was more frequently observed in patients without a diagonal branch flow than in patients with a diagonal branch flow (49.3% vs. 18.8% in II, *p* < .01; 66.4% vs. 36.3% in III, *p* < .01; 63.7% vs. 30.0% in aVF, *p* < .01, respectively). Hence, those with a diagonal branch flow showed a higher proportion of patients with a baseline ST‐segment level in these leads since the presence of a diagonal branch flow allows it to elevate to a baseline level (50.0% vs. 78.8% in II; 32.9% vs. 63.8% in III; 35.6% vs. 68.8% in aVF, respectively). With a wrapped LAD, an ST‐segment elevation in leads II, III, and aVF was characteristic findings in patients with a diagonal branch flow compared to patients without a diagonal branch flow (50.0% vs. 0% in II, *p* < .01; 60.0% vs. 0% in III, *p* < .01; 60.0% vs. 0% in aVF, *p* < .01, respectively).

**Table 3:** ST‐segment deviation: LAD morphology and diagonal branch flow

ST deviation, *n*	Wrapped LAD, *n* = 26							Nonwrapped LAD, *n* = 226						
	Diagonal br. flow −, *n* = 16			Diagonal br. flow +, *n* = 10			*p* Value	Diagonal br. flow −, *n* = 146			Diagonal br. flow +, *n* = 80			*p* Value
	**↑**	**→**	**↓**	**↑**	**→**	**↓**		**↑**	**→**	**↓**	**↑**	**→**	**↓**	
I	3	12	1	2	8	0	.72	53	91	2	12	66	2	<.01
II	0	11	5	5	5	0	<.01	1	73	72	2	63	15	<.01
III	0	10	6	6	4	0	<.01	1	48	97	0	51	29	<.01
aVR	0	16	0	0	9	1	.20	12	126	8	3	77	0	.04
aVL	5	11	0	1	9	0	.21	74	72	0	13	66	1	<.01
aVF	0	9	7	6	4	0	<.01	1	52	93	1	55	24	<.01
V_1_	8	8	0	6	4	0	.62	97	49	0	46	34	0	.18
V_2_	16	0	0	10	0	0	N/A	146	0	0	80	0	0	N/A
V_3_	16	0	0	10	0	0	N/A	146	0	0	80	0	0	N/A
V_4_	14	2	0	8	2	0	.61	132	12	2	72	8	0	.53
V_5_	12	4	0	6	4	0	.42	101	42	3	49	30	1	.38
V_6_	6	10	0	3	6	1	.43	43	83	20	17	58	5	.05

Abbreviation: LAD, left anterior descending artery.

In leads Ⅰ and aVL, a higher proportion of patients without a diagonal branch flow showed ST‐segment elevation than patients with a diagonal branch flow with a nonwrapped LAD (36.3% vs. 15.0% in Ⅰ, *p* < .01; 50.7% vs. 16.3% in aVL, *p* < .01, respectively). However, this difference was not observed for wrapped LAD when comparing patients with and without a diagonal branch flow (18.8% vs. 20.0% in Ⅰ, *p* = .72; 31.3% vs. 10.0% in aVL, *p* = .21, respectively).

### 3.3 Diagnostic flowchart to estimate the morphology of LAD lesions

**Figure 1:**
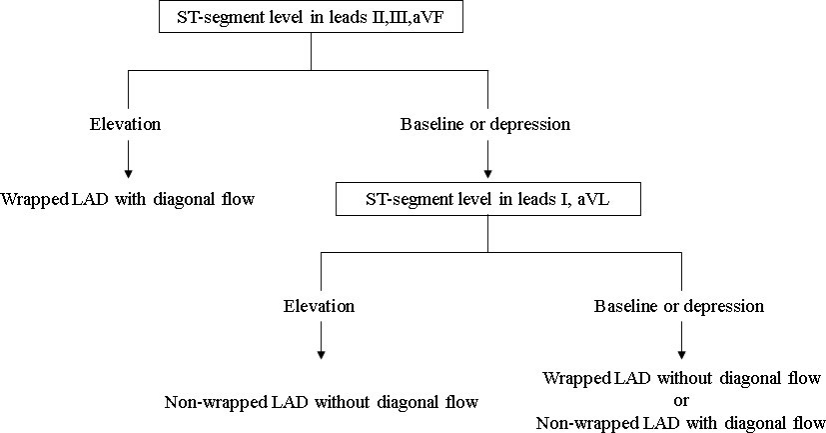
Diagnostic flow diagram to estimate LAD lesions. When the ST‐segment elevation is shown in all of leads II, III, and aVF, and the lesion is characterized is a “wrapped left anterior descending artery (LAD) with diagonal branch flow,” the diagnostic accuracy when had a sensitivity of 0.500, specificity of 1.000, positive predictive value (PPV) of 1.000, and negative predictive value (NPV) of 0.980, respectively. When the ST‐segment elevation is shown in both leads Ⅰ and aVL after failing the above first step, the diagnostic accuracy of a “nonwrapped LAD without diagonal branch flow” has a sensitivity of 0.342, specificity of 0.871, PPV of 0.794, and NPV of 0.478, respectively. ST‐segment deviation alone cannot be used to decide whether lesion morphology is a wrapped LAD without a diagonal branch flow or a nonwrapped LAD with a diagonal branch flow

Figure 1 shows a suggested diagnostic flowchart for estimating the morphology and location of culprit LAD lesions according to the distribution of ST‐segment deviations. When a ST‐segment elevation is observed in all of leads II, III, and aVF, the diagnostic accuracy when the lesion was characterized as a “wrapped LAD with a diagonal branch flow” showed a sensitivity of 0.500, specificity of 1.000, PPV of 1.000, and NPV of 0.980, respectively. When an ST‐segment elevation is observed in both leads Ⅰ and aVL in patients who do not satisfy the above criterion, the diagnostic accuracy when the lesion is characterized as a “nonwrapped LAD without a diagonal branch flow” showed a sensitivity of 0.342, specificity of 0.871, PPV of 0.794, and NPV of 0.478, respectively. In comparison, a statistical difference in ST‐segment deviation across all 12 leads was not found between a “wrapped LAD without diagonal branch flow” and a “nonwrapped LAD with diagonal branch flow” (Figure S1, S2, S3, and S4). The QTc, both Bazett's and Fridericia's, was the sole electrocardiographic parameter to show a statistical difference between a “wrapped LAD without diagonal branch flow” and a “nonwrapped LAD with diagonal branch flow” as shown in Table 4 (Bazett's, 467.8 [452.0, 483.8] vs. 437.9 [425.1, 467.2], *p* = .01; Fridericia's, 444.3 [420.9, 460.7] vs. 427.0 [412.6, 443.0], *p* = .04, respectively).

**Table 4:** Comparison between wrapped LAD without diagonal branch flow and nonwrapped LAD with diagonal branch flow

	Wrapped LAD without diagonal br. flow	Nonwrapped LAD with diagonal br. flow	*p* Value
HR, bpm	80.5 (72.3, 106.0)	78.0 (62.3, 89.8)	.16
QRS axis, degree	19 (−29.8, 62.0)	38.5 (−1.8, 58.8)	.39
QRS duration, ms	92.0 (86.0, 101.5)	95 (86.0, 104.0)	.62
LAHB, *n*	2 (12.5%)	2 (2.5%)	.07
QT interval, ms	404.5 (363.0, 415.5)	393 (374.5, 422.0)	.93
QTc, Bazett	467.8 (452.0, 483.8)	437.9 (425.1, 467.2)	.01
QTc, Fridericia	444.3 (420.9, 460.7)	427.0 (412.6, 443.0)	.04

Abbreviations: HR, heart rate; LAHB, left anterior hemiblock; QTc, corrected QT.

## 4 DISCUSSION

The present study assessed the relationship between the distribution of ST‐segment deviations, and the morphology and location of culprit LAD lesions by initial ECG in STEMI. We designed a diagnostic flowchart to estimate the morphology and location of culprit LAD lesions. ST‐segment elevation in leads Ⅰ, aVL, and V_1_ was frequent in patients with the LAD lesion proximal to the first septal branch. An ST‐segment elevation in inferior leads was characteristic of a wrapped LAD with a diagonal branch flow, but the loss of a diagonal branch flow canceled this elevation. For a nonwrapped LAD, a ST‐segment elevation in lateral leads was more frequent in lesions proximal rather than distal to the diagonal branch, but this finding was not observed in patients with a wrapped LAD. Although it is difficult to distinguish between a wrapped LAD without a diagonal branch flow and a nonwrapped LAD with a diagonal branch flow because of the homology of the ST‐segment deviation between these, the QTc interval was the sole electrocardiographic parameter showing a significant difference between the two groups. Taking these findings into consideration, the present study puts forward a diagnostic flowchart to estimate such anatomical features. This was confirmed to have reasonably good diagnostic accuracy.

### 4.1 Role of diagonal branch in reciprocal ST‐segment depression

Acute transmural ischemia provides incomplete depolarization and decreases the resting membrane potential. This leads to a potential difference from normal regions and causes ST‐segment elevation in the leads corresponding to ischemic regions. For the electrode directly under the ischemic region, a relatively increased membrane potential and plateau potential make a reciprocal ST‐segment depression in the opposite leads. Acute LAD occlusion can lead to an ST‐segment elevation in precordial leads caused by ischemia in the anteroseptal area and a ST‐segment depression in the inferior leads as a reciprocal change. A reciprocal ST‐segment depression is not always observed in all cases of LAD occlusion. The present study demonstrated that a diagonal branch flow has an important role in forming the reciprocal change.

The loss of this flow causes broad and severe ischemic changes in the high lateral and anterior myocardium, and it leads to a major potential difference between a broadly ischemic myocardium in the anterior area and a viable myocardium in the inferior area; this major potential difference provides a significant reciprocal ST‐segment depression in inferior leads.

### 4.2 The particularity of wrapped LAD

Although the above statement regarding the relationship between the diagonal branch flow and reciprocal ST‐segment depression applies for the typical morphology of a nonwrapped LAD, a wrapped LAD was found in 10.3% of the present study population, which had different characteristic findings from those of a nonwrapped LAD. The acute occlusion of a wrapped LAD, which perfuses the inferior area through the cardiac apex, showed an ST‐segment elevation in the inferior leads in addition to that in precordial leads. A preserved diagonal branch flow increases the potential difference between viable anterior and ischemic inferior areas and makes a significant ST‐segment elevation in inferior leads. A loss of flow offsets the potential difference and this elevation becomes inconspicuous (Sasaki et al., 2001). The present study was able to clearly address these mechanisms.

### 4.3 Impact of septal and diagonal branch flows

The present study demonstrated LAD lesions proximal to the first septal branch showed an ST‐segment elevation in leads Ⅰ, aVL, and V_1_. Several specific ECG findings which suggest lesions proximal to the first septal branch have been reported, such as an ST‐segment elevation in lead aVR, an ST‐segment depression in lead V_5_, and an ST‐segment elevation in lead V_1_ (Birnbaum et al., 1994; Engelen et al., 1999). Since it is considered that a lead V_1_ reflects a ischemic change at the basal septal wall (Ben‐Gal et al., 1998), an ST‐segment elevation in lead V_1_ is a well‐known finding of the lesion proximal to the septal branch. However, this has been controversial in several reports about its clinical confirmation (Ben‐Gal et al., 1997; Birnbaum et al., 1994; Engelen et al., 1999). In anatomical perspective, lead V_1_ reflects the right paraseptal area supplied by the septal branches. A septal myocardium is supplied by both septal branch and conus branch, and the dominancy between these branches might determine whether septal branch occlusion leads ST‐segment elevation in V_1_ (Ben‐Gal et al., 1997). The present study suggested that the criterion, including an ST‐segment elevation ≥0.1 mV in lead V_1_, is a finding which has low sensitivity but high specificity for a proximal lesion to the first septal branch. We must be careful in interpreting these results since lack of anatomical physiological confirmation (Allencherril et al., 2018a).

The present study demonstrated a ST‐segment elevation, in lateral leads, Ⅰ and aVL, in lesions proximal to a diagonal branch and identical to lesions proximal to the septal branch since most diagonal branches are from the same level or distal to the first septal branch. However, a ST‐segment elevation in lead V_1_ was not a specific finding. A ST‐segment elevation in lateral leads is thought to be derived from ischemia in the high lateral wall that is related to the involvement of the first diagonal branch (Arbane & Goy, 2000; Birnbaum et al., 1993). The present study demonstrated this finding only for nonwrapped LAD and could not be shown for wrapped LAD. Sasaki et al. (2001) reported that an ST‐segment elevation in lateral leads caused by ischemia in this area is often offset by reciprocal changes caused by ischemia in the inferior wall due to the occlusion of a wrapped LAD. A statistical difference was not found in lateral leads between lesions proximal and distal to the diagonal branch in a wrapped LAD demonstrated in our study. This is thought to be a result of the ischemia at the inferior wall due to the occlusion of a wrapped LAD that canceled the ST‐segment elevation in lateral leads as suggested by Sasaki et al. (2001). Allencherril et al. (2018b) reported that the concomitant apical inferior ischemia attenuated ST elevation in lead aVL using cardiac magnetic resonance imaging. The present study referred to the report by Sasaki et al. (2001), and their report had a great contribution to establish our diagnostic flow.

### 4.4 Difference between wrapped LAD without diagonal branch flow and nonwrapped LAD with diagonal branch flow

A loss of diagonal branch flow offset the ST‐segment elevation in inferior leads in wrapped LAD, and a preserved diagonal branch flow offset the reciprocal ST‐segment depression in nonwrapped LAD. Hence, a significant difference was not observed in the ST‐segment deviation in inferior leads between these two populations, and a ST‐segment elevation in lateral leads was also not observed in both populations due to the above. As a result, these two populations did not show a difference in the overall distribution of the ST‐segment deviation through all limb leads, making it difficult to differentiate between these two populations on this basis.

A fine difference may potentially exist between these two populations because of a difference between the canceled elevation and depression. Although the present study adopted the criterion of a ST‐segment depression in limb lead as ≥0.1 mV based on AHA/ACCF/HRS recommendations (Wagner et al., 2009), measurements using a finer scale may allow this minor difference to be made clearer. However, these two populations should show a major difference in infarct size (Kobayashi et al., 2015). When using electrocardiographic parameters other than an ST‐segment deviation to differentiate them, the QTc interval was suggested as a sole identifiable parameter. The QTc interval is well known as a sensitive parameter which reflects infarct size (Raev, 1997; Stajer, Mozina, Noc, & Rode, 1993; Tamura, Nagase, Mikuriya, & Nasu, 1999).

### 4.5 Diagnostic flow to estimate anatomical features

The present study suggested that diagnostic flow could be used to estimate anatomical characteristics. The diagnostic accuracy was confirmed to be comparatively high in spite of the simple algorism. However, we are unable to distinguish between a wrapped LAD without diagonal branch flow and a nonwrapped LAD with diagonal branch flow by this flow diagram only. Other electrocardiographic parameters, including a QTc interval or other diagnostic modalities, are necessary.

The present study has several limitations. A preceding ECG before STEMI onset was not considered in the analysis since STEMI is not a scheduled event. In addition, several baseline characteristics may possibly impact ECG findings. Finally, the sample size of patients with a wrapped LAD was small.

## 5 CONCLUSION

An initial ECG in STEMI is useful to estimate culprit LAD morphology or the existence of major side branch flow.

## CONFLICT OF INTEREST

The authors declare that they have no conflicts of interest.
