# Supplementary figures and images for: Diagnostic flowchart to estimate the morphology of left descending artery lesions by initial electrocardiogram in ST‐elevation myocardial infarction
Source: Ann Noninvasive Electrocardiol. 2019 Sep 25;25(1):e12695. doi: 10.1111/anec.12695 (PMC7358834; doi:10.1111/anec.12695)

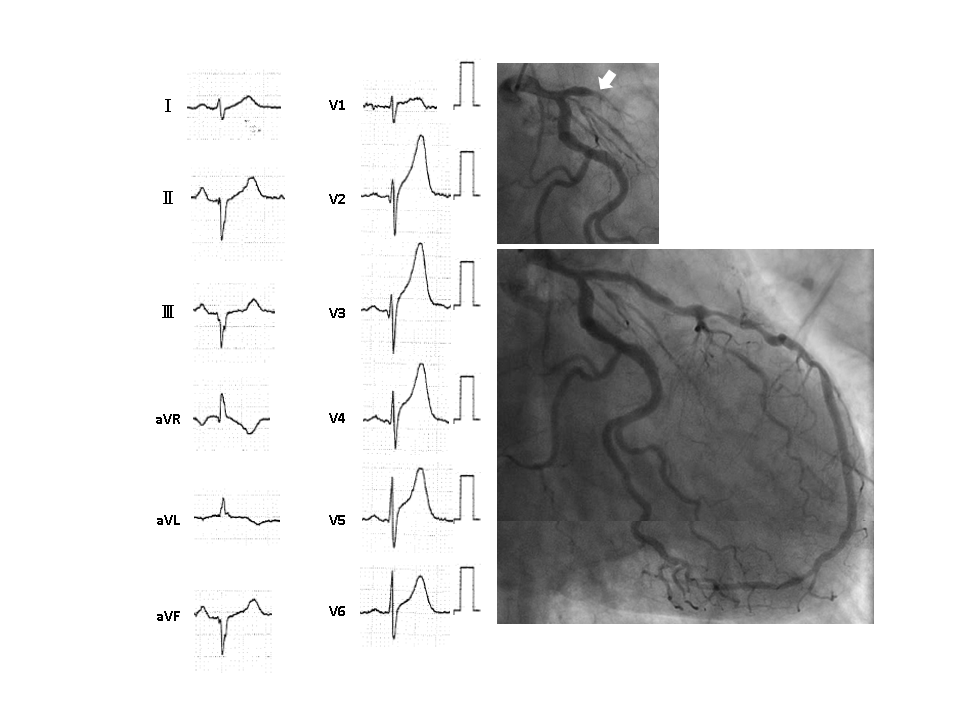

Supplement: Supplementary file 1 [file ANEC-25-e12695-s001.TIF]

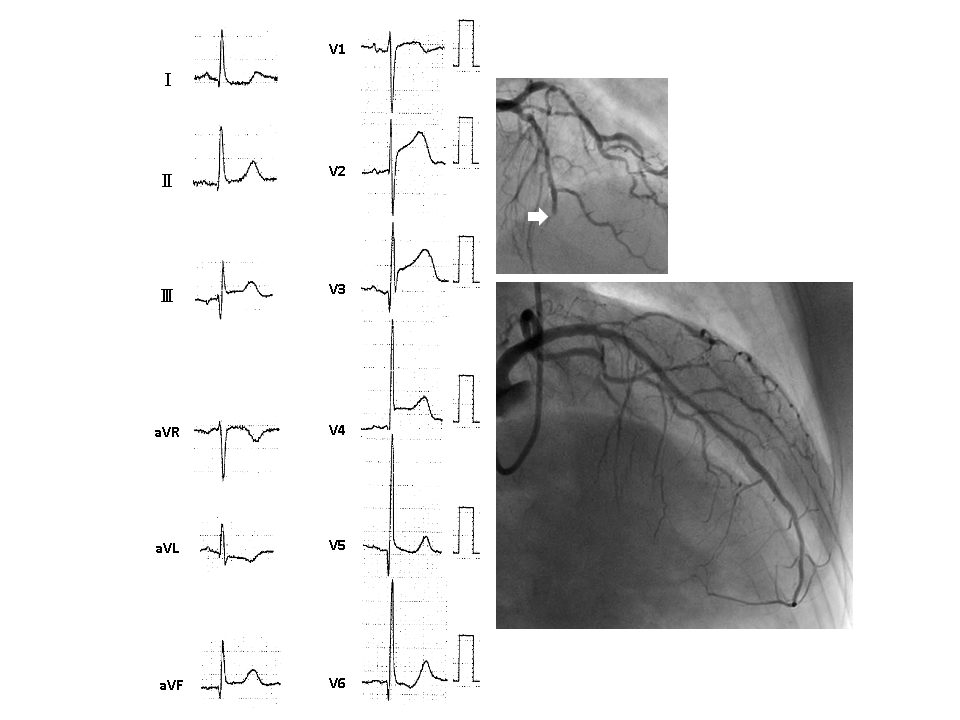

Supplement: Supplementary file 2 [file ANEC-25-e12695-s002.TIF]

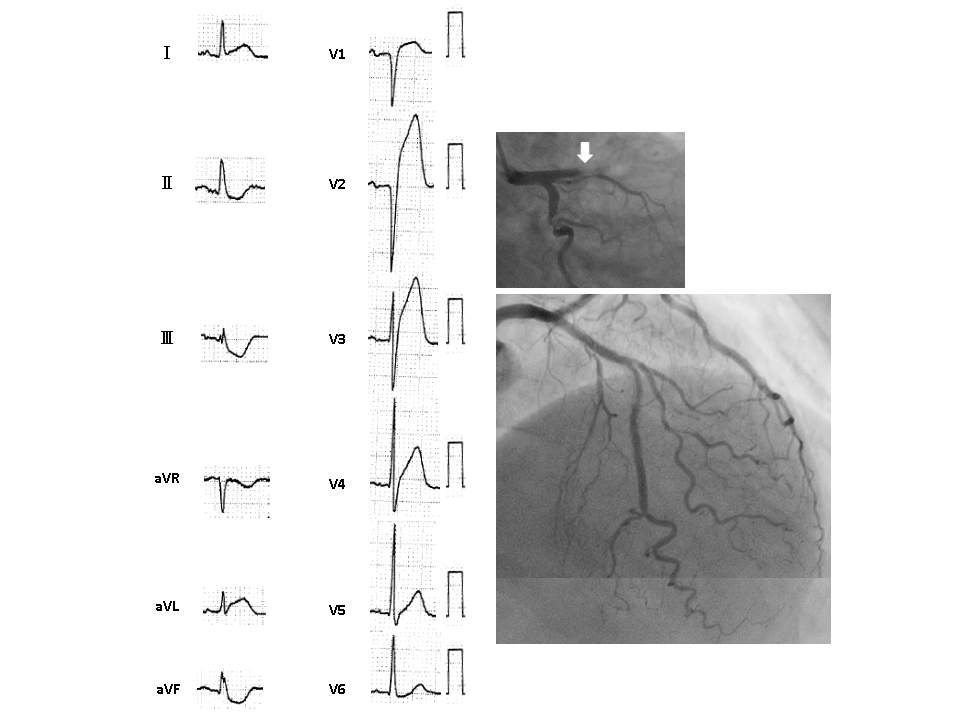

Supplement: Supplementary file 3 [file ANEC-25-e12695-s003.TIF]

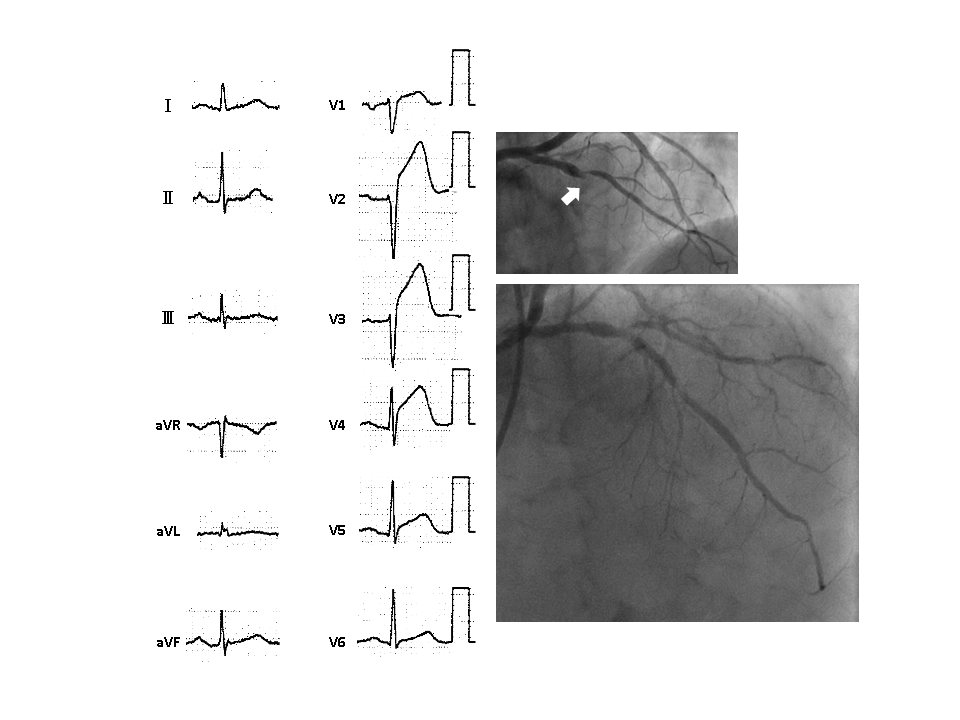

Supplement: Supplementary file 4 [file ANEC-25-e12695-s004.TIF]
